# Polyphenol metabolomics reveals the applications and prospects of polyphenol-rich plants in natural dyes

**DOI:** 10.48130/forres-0024-0035

**Published:** 2024-12-19

**Authors:** Jing Gao, Yunxiao Zhao, Feifei Ni, Ming Gao, Liwen Wu, Zhicheng Yu, Yicun Chen, Yangdong Wang

**Affiliations:** 1 State Key Laboratory of Tree Genetics and Breeding, Chinese Academy of Forestry, Beijing 100091, China; 2 Nanjing Forestry University, Nanjing 210037, Jiangsu Province, China; 3 Research Institute of Subtropical Forestry, Chinese Academy of Forestry, Hangzhou 311400, Zhejiang Province, China; 4 Zhejiang Sci-Tech University, Hangzhou 310018, Zhejiang Province, China

**Keywords:** Polyphenol metabolomics, Naringenin, Tannin, Natural dyes

## Abstract

Polyphenols, as one of the primary compounds produced by plant secondary metabolism, have garnered considerable attention because of their non-toxic, environmentally friendly, and biodegradable properties, as well as their notable medicinal value. This study presents a metabolomic analysis of polyphenols from 11 woody plants, including *Camellia oleifera*, *Quercus acutissima*, and *Punica granatum*, investigating a total of 40 polyphenolic metabolites. A differential metabolite dynamics map highlighted the five most differentiated substances among the 11 plants, including vitexin, dihydromyricetin, genistin, resveratrol, and isorhamnetin. To evaluate the application of polyphenol-rich plants as natural dyes, dye performance tests, and color fastness evaluations were conducted, focusing on the specific role of polyphenols in dyeing cotton fabrics. The composition of polyphenols had a minor effect on the color of dyed cotton fabrics, typically imparting only black or brown tones to the fabric. However, their effect on dyeing performance is notable, with the ratio of the dye absorption coefficient (k) to the dye scattering coefficient (s) (K/S) ranging from 1 to 20, and lightness varying from 26 to 78. The addition of mordants not only improved the dye's color fastness but also expanded the color range. Furthermore, this study identified four key substances that influence the dyeing performance of plant dyes, including naringenin, epicatechin, catechin, and dihydromyricetin, and discovered a novel natural dye compound, naringenin. Importantly, six of the 11 plant dyes selected in this study are derived from plant waste, thus providing a theoretical basis for advancing environmentally friendly and sustainable dyeing technologies.

## Introduction

Plant polyphenols, also known as vegetable tannins, are large molecular compounds widely present in the roots, bark, stems, leaves, flowers, fruits, and peel of plants, with their content being second only to cellulose, hemicellulose, and lignin^[1]^. These compounds can be categorized into five groups based on their structural formulas and chemical structures: tannins, flavonoids, coumarins, phenolic acids, and stilbenes^[2,3]^. Generally, polyphenols are extracted from plants using methods such as solvent extraction, ultrasonic extraction, and microwave extraction^[4,5]^. Due to their antioxidant, antibacterial, and antiradiation properties, these compounds have been applied widely in food, pharmaceuticals, and textile dyeing and printing^[6−9]^. During dyeing, polyphenol compounds generally exhibit three colors: gray, dark brown, and black^[10]^. Importantly, certain polyphenol compounds contain phenolic hydroxyl, hydroxyl, and carboxyl groups that, when in contact with metal mordant, can form new metal complexes^[11]^. These complexes can effectively improve dye fixation.

Natural dyes are pigments extracted from natural sources and can be utilized for coloring textiles, food, and cosmetics. Their primary sources include plants, animals, minerals, and microorganisms. Among these, plant dyes are the most prevalent in the natural dye market because of their availability, natural origin, eco-friendliness, health benefits, and wide range of color options^[12]^. These dyes are extracted from various plant parts, including flowers, leaves, roots, stems, and fruits. Plant dyes contain different kinds of molecular structures, which can show a variety of colors, including polyphenols, carotenoids, naphthoquinones, anthraquinones, flavonoids, indigo, chlorophyll, etc. Common examples include safflower, madder, logwood, and gardenia^[13,14]^.

The methods for extracting plant dyes include solvent extraction, supercritical fluid extraction, and auxiliary extraction techniques such as ultrasound, microwave, and enzymatic extractions^[15]^. Based on whether mordants are added during the dyeing process and the type of dye used, dyeing techniques can be categorized into direct dyeing, mordant dyeing, and over-dyeing^[16]^. Within mordant dyeing, depending on the sequence in which the mordant and dye are applied, the mordanting process can be further divided into the following methods: simultaneous mordanting, pre-mordanting, post-mordanting, and multiple mordanting^[17,18]^. Metal ion mordants are the most commonly used mordants in dyeing processes that enable dye molecules to form complex bonds with the fabric fibers, thus altering the hue and achieving color fixation. Common metal ion mordants include ferrous sulfate, alum, and copper acetate^[19,20]^.

Currently, most research on natural dyes focuses on their dyeing applications. Natural fibers can be classified into cellulose and protein fibers, with cotton being the most widely used natural fiber^[21,22]^, and its dyeing and functional modification have considerable implications for other natural fibers^[23]^. A study on the adsorption characteristics, thermodynamic parameters, and kinetics of *Scutellaria orientalis* water extract for dyeing cotton fabric without mordanting provides foundational dyeing performance data for future research on natural dyes^[24]^. Using chitosan as a mordant in combination with lotus seed dye for dyeing cotton fabric enhances dyeing quality, color fastness, and antimicrobial activity, demonstrating the great potential of chitosan in developing sustainable antimicrobial textiles^[25]^. The use of natural dyes faces considerable wastewater pollution issues, research has shown that using aluminum sulfate as a mordant can minimize the pollution caused by natural dyes during the cotton dyeing process^[26]^. With improvements in living standards, natural dyes have been increasingly applied to other natural fibers such as silk and lyocell while also integrating artificial intelligence to advance the smart development of dyeing processes^[27,28]^.

The widespread application of plant dyes is hindered by several challenges, including unstable dyeing performance, difficulties in color blending, uneven coloration, poor durability, complex dyeing processes, and high costs^[29,30]^. The vast majority of natural dyes are derived from plants. The content and composition of pigments can be influenced by various factors, including the geographical origin of plants, differing climatic conditions, and harvest timing. These variables contribute to the inconsistent dyeing results achieved with plant-based dyes, complicating standardization and hindering mass production. Additionally, the effective utilization rate of vegetable dyes remains low, leading to high dyeing costs. Moreover, the dye solutions extracted from plants are typically mixtures, lacking a specific component that governs dyeing performance, considerably restricting the diverse applications of plant dyes^[31]^.

This study selected 11 polyphenol-rich plants as research materials^[32−34]^, including *Camellia oleifera* shells, *Quercus acutissima* shells, *Punica granatum* peels, *Diospyros kaki*, *Galla Chinensis*, *Dioscorea cirrhosa*, *Camphora officinarum* seeds, *Juglans regia* shells, *Fallopia multiflora*, *Vernicia fordii* shells, and *Castanea mollissima* shells. The tannin content and polyphenolic metabolites were tested to explore further the differences among these plants and their potential dyeing capabilities. Plant dyes were extracted from these materials using microwave extraction with water and 60% anhydrous ethanol as solvents, resulting in dyes with varying compositions^[35]^. Three dyeing methods were used to dye cotton fabric: direct dyeing, post-mordanting with ferrous sulfate, and post-mordanting with alum. This study aims to systematically analyze polyphenol-rich plants and their effects on the dyeing performance of cotton fabrics, filling gaps in the analysis of individual components of polyphenolic natural dyes. It provides new insights and strategies for the green and eco-friendly application of natural dyes, advancing the sustainable development of the plant dye industry.

## Materials and methods

### Materials and chemicals

The 11 plant materials were collected or purchased by the research team in the Fuyang field of Hangzhou City, China (30°27'94" N, 119°58'43" E). Specifically, the *C. oleifera* shells, *Q. acutissima* shells, *P. granatum* peels, *C. camphora* seeds, *J. regia* shells, *V. fordii* shells, and *C. mollissima* shells were collected locally, dried, and stored. The remaining four plants, *D. kaki*, *G. Chinensis*, *D. cirrhosa*, and *F. multiflora* were purchased from the local market. All the chemical reagents used in the experiment were of laboratory grade. The pure cotton knit fabric used in this experiment has an area weight of 200 g/m^2^.

### Determination of tannin content

Tannins can reduce ferric ammonium citrate in an alkaline solution, forming a deep blue compound, the intensity of which is proportional to the tannin content. This can be quantified by comparing the results with a standard curve. For the 11 plant materials mentioned above, after grinding, 0.1 g of each sample was placed into a 15 mL test tube. To this, 1 mL of dimethylformamide solution was added, followed by shaking for 60 min and centrifugation at 4,000 rpm for 10 min. A 0.25 mL aliquot of the supernatant was then taken, followed by the addition of 1.5 mL of deionized water (ddH_2_O) and 0.25 mL of ammonia solution. The mixture was shaken, left to stand at room temperature for 10 min, and the absorbance at 525 nm was measured as A1. In the second test tube, 0.25 mL of the extract was added to 1.25 mL ddH_2_O, 0.25 mL ammonia solution, and 0.25 mL ferric ammonium citrate solution. After shaking and standing for 10 min, the absorbance at 525 nm was measured as A2. The tannin content was quantitatively analyzed using a Multiskan GO microplate spectrophotometer (Thermo, USA). The tannin absorbance measurement was calculated as A = A2 − A1. The tannin content in the sample was then determined based on the standard curve of gallic acid.

### Targeted metabolic detection of polyphenols

An appropriate amount of sample was placed into a centrifuge tube, and 80% methanol aqueous solution (containing 0.2% vitamin C) was added. The mixture was subjected to ultrasonic extraction at room temperature for 30 min, followed by centrifugation at 12,000 rpm for 10 min to collect the supernatant. This extraction process was repeated twice, and the supernatants from both extractions were combined and mixed. The resulting solution was then diluted several times as needed before further analysis. Metabolite detection in samples was performed using ultra-high-performance liquid chromatography coupled with tandem high-resolution Orbitrap mass spectrometry (UHPLC-QE, Thermo, USA). Qualitative and quantitative analysis of polyphenolic metabolites in the 11 plant species was conducted by comparing the retention time and molecular mass (mass error <10 ppm) with those of the standards^[34]^.

### Data analysis

Clustering analysis, principal component analysis (PCA), and orthogonal partial least squares discriminant analysis (OPLS-DA) were employed to observe the overall distribution and stability of the samples, as well as to distinguish metabolic differences between groups and identify differential metabolites. The criteria for selecting differential metabolites between groups were VIP > 1 from the first principal component of the OPLS-DA model and *p* < 0.05 from the Student's *t*-test.

### Extraction of natural plant dyes

Microwave penetration is strong and selective heating is efficient, resulting in higher extraction efficiency compared to other methods. Therefore, microwave-assisted extraction (MAE) was chosen to obtain plant dyes from 11 types of plants^[36]^. Water and 60% ethanol were used as solvents to extract different plant dye components^[35,37]^. After drying, the plant samples were ground in a grinder, and a certain amount of powder was weighed. The powder was then added to water or 60% ethanol solution at a material : liquid ratio of 1:20. MAE was performed at 60 °C for 60 min. After extraction, the solution was filtered to remove the residues, and the resulting dye solution was stored at 4 °C^[38]^. After extraction, the solution was filtered to remove the residues. The resulting solution was stored at 4 °C for subsequent dyeing.

### Dyeing process

**Figure 1:**
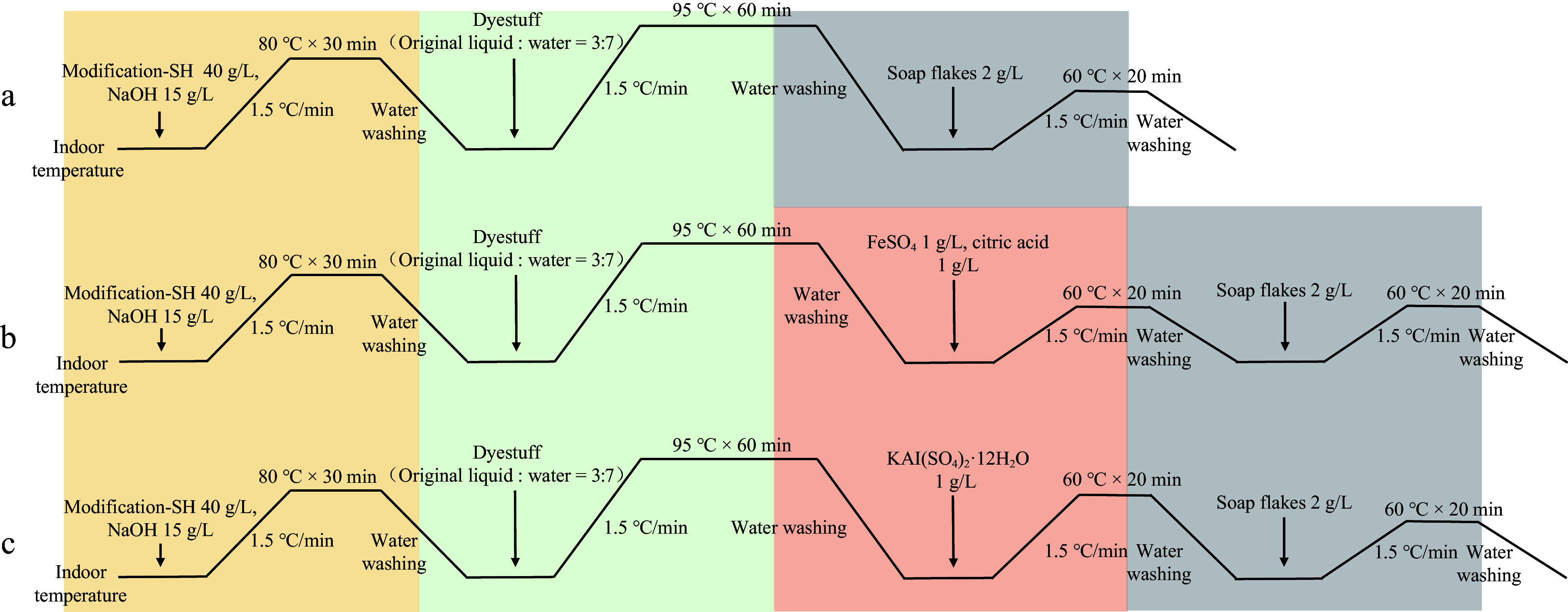
Dyeing process flow chart for plant dyes. (a) Direct dyeing process flow with plant dyes. (b) Post-mordanting process flow with FeSO_4_ as a mordant for plant dyes. (c) Post-mordanting process flow with KAl(SO_4_)_2_·12H_2_O as a mordant for plant dyes. SH refers to thiol-based modifiers.

In all dyeing processes, the liquor ratio of the cotton fabric to the solution was 1:50, meaning 2 g of cotton fabric was placed in 100 mL of different solutions for the reaction. Firstly, the modification process starts at room temperature, with the temperature being increased at a rate of 1.5 °C/min to 80 °C, maintained for 30 min, then cooled and washed with water. Since the post-mordanting method was chosen for dyeing the cotton fabric, the fabric is first dyed. The dye solution is prepared with a ratio of dye stock solution to water at 3:7. Starting at room temperature, the temperature is increased at a rate of 1.5°C/min to 95 °C, maintained for 60 min, then cooled and washed with water. In the direct dyeing method, after dyeing, the cotton fabric is directly subjected to soaping and drying for subsequent experiments. For fabrics to be mordanted with FeSO_4_ or KAl(SO_4_)_2_·12H_2_O, a mordant solution was prepared after dyeing. Starting at room temperature, the temperature is increased at a rate of 1.5 °C/min to 60 °C, maintained for 20 min, then cooled and washed with water, followed by soaping and drying (Fig. 1)^[39,40]^.

### Dyeing performance testing

Under the D65 light source and a 10° viewing angle, the K/S value was calculated using the SF600 PLUS computer colorimeter (Datacolor, USA) based on the Kubelka-Munk equation: K/S = (1 − R)^2^/2R, where R is the reflectance^[41]^. The K/S value reflects the color strength of the dye on cotton fabric. A higher K/S value indicates greater depth and intensity of the color. Each sample was measured three times, and the average was taken to ensure accuracy. The color values were expressed using CIE Lab coordinates, including lightness (L* ), red/green coordinate (a*), and yellow/blue coordinate (b*), to quantify the color performance of the dyed samples. The color differences between the dyed cotton fabric samples and the standard reference were calculated using the CIE Lab color system. L*: Represents differences in lightness or darkness; larger values indicate brighter colors; a*: Reflects red and green tonal differences; higher values indicate redder tones; b*: Indicates yellow and blue tonal differences; higher values suggest yellower tones; c*: Represents differences in color saturation; positive values indicate more vivid colors, while negative values denote duller tones; h*: Reflects variations in hue. For each sample, five random points were measured during testing and the average value was taken as the result^[42]^.

### Color fastness evaluation

The color fastness characteristics of the dyed fabrics are assessed according to Chinese Textiles Test Specifications for Color Fastness based on ISO international standards. Color fastness to washing (GB/T 3921-2008), and to light (GB/T 8427-2019).

### Surface analysis of dyed cotton fabrics

The surface of the dyed fabric was examined using a scanning electron microscope (SEM) (Phenom, Phenom ProX, The Netherlands) to analyze the effect of the dye on the cotton fabric. The fabric is placed on the sample stage and coated with gold before observing the surface morphology of the dyed fabric under an accelerating voltage of 10 kV.

## Results

### Polyphenolic metabolite analysis reveals unique coloring properties associated with plant dyes

**Figure 2:**
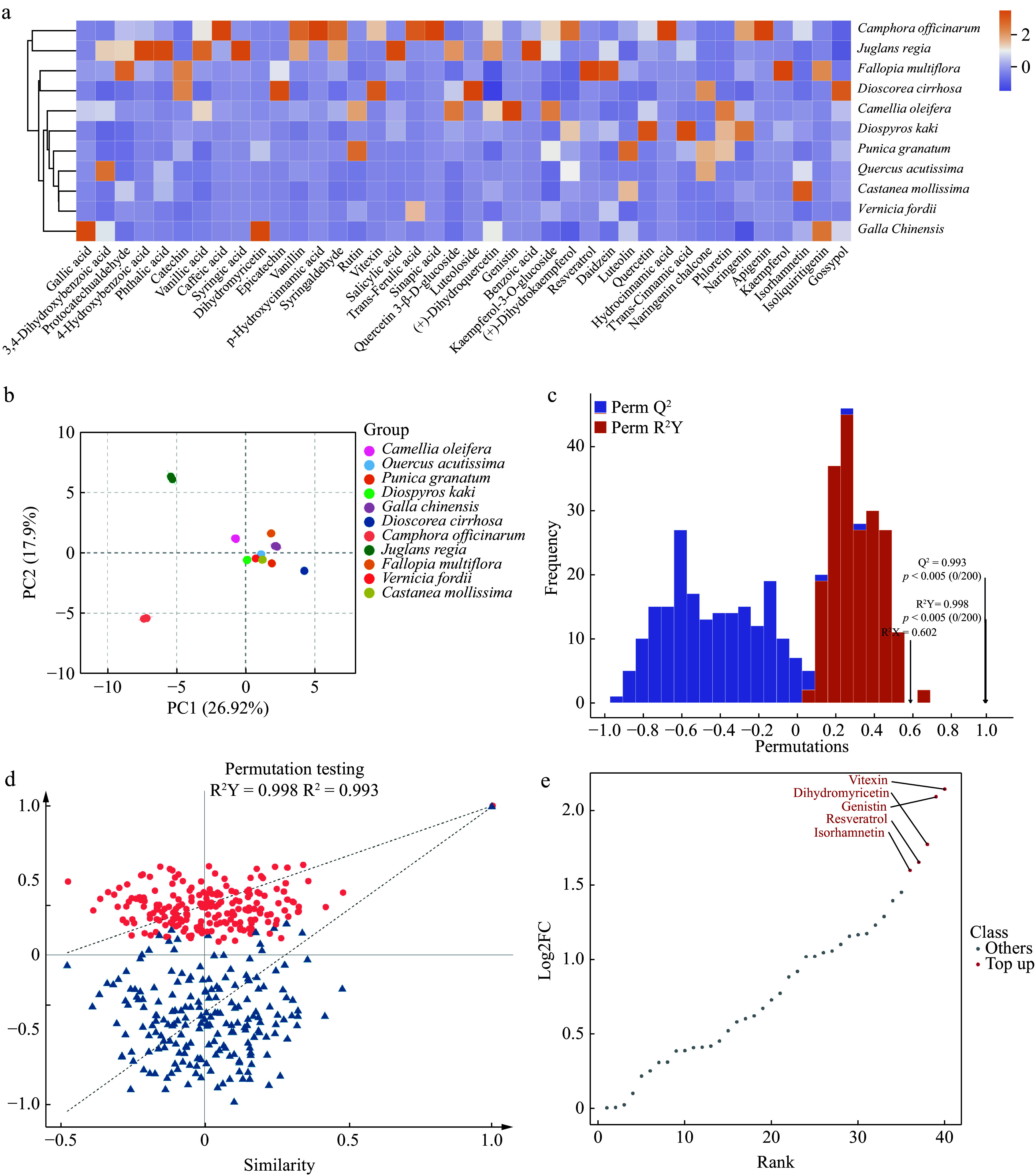
Analysis of polyphenolic metabolites in 11 plants. (a) Heatmap of polyphenolic compound content, showing the relative content of polyphenolic compounds in 11 plants. The color gradient reflects the distribution differences in metabolites among plants, with darker colors indicating higher contents. (b) Principal component analyses (PCA) of polyphenolic metabolites in 11 plants. PC1 indicates the first principal component, PC2 indicates the second principal component, and the percentage indicates the explanation rate of this principal component to the data set. PCA score plot, clearly revealing the clustering characteristics and differences in polyphenolic metabolites across 11 plants based on the distribution of PC1 and PC2. (c) OPLS-DA model validation plot, demonstrating the model's high explanatory power (R²Y) and excellent predictive performance (Q²) for polyphenolic metabolite data. (d) OPLS-DA permutation test plot, confirming the absence of overfitting and further validating the statistical reliability of this model. (e) Differential metabolite dynamics map, highlighting the variation patterns of the five most considerably different polyphenolic metabolites among the 11 plants.

The different polyphenol components in plants endow them with unique dyeing properties. First, a metabolomic analysis of the polyphenols in 11 plant species was conducted. A total of 40 polyphenolic metabolites were detected across the 11 plant materials. After normalizing these data, a cluster heatmap (Fig. 2a) was generated, clearly demonstrating differences in the contents of these compounds in different plants. Notably, the polyphenolic metabolites in *J. regia* shells and *C. officinarum* seeds differed considerably from those in the other nine plants, exhibiting a unique diversity. Additionally, the gallic acid and dihydromyricetin contents in *G. chinensis* were significantly higher than in the other groups, demonstrating a markedly different metabolic pattern compared with the other plants.

To further ensure reliable, reproducible, and high-quality metabolomic data, principal component analysis (PCA) was conducted on three quality control (QC) samples from each of the 11 different plants (Fig. 2b)^[43]^. The first and second principal components (PC1 and PC2) account for 26.92% and 17.9% of the variance in these data, respectively, indicating notable differences in the polyphenol metabolites among the various plants. The results demonstrate that PCA can distinguish between different plants and QC samples. More importantly, the PCA results are consistent with the clustering analysis. Except for *J. regia* shells and *C. officinarum* seeds, the PCA distributions of polyphenol metabolites from the other nine plants are relatively close, suggesting similar polyphenol metabolic profiles. OPLS-DA was used to compare the polyphenols in the 11 plant samples. The OPLS-DA model shows a Q² > 0.9 (0.993), indicating an excellent model, while the R²Y value is close to 1 (0.998) and signifies a reliable explanation rate for the Y matrix by the model. The OPLS-DA permutation test also confirms that the permutation test passed, and the model does not exhibit overfitting (Fig. 2c & d); therefore, notable differences exist among the polyphenol metabolomes of the different plants, allowing for the screening of different metabolites based on the variable importance in projection values. Polyphenols with considerable differences among the various plants are indicated based on their distance from the origin. Differential metabolite dynamics maps show the five most differentiated substances vitexin, dihydromyricetin, genistin, resveratrol, and isorhamnetin among the 11 plants (Fig. 2e). The polyphenol metabolome analysis and screening of differential metabolites for different plant dyes can help reveal their unique coloring properties and stability during the dyeing process, thus providing a scientific basis for developing efficient and environmentally friendly natural dyes.

### Different solvents and mordants considerably affect the dyeing performance and color range of polyphenol-rich plant dyes

**Figure 3:**
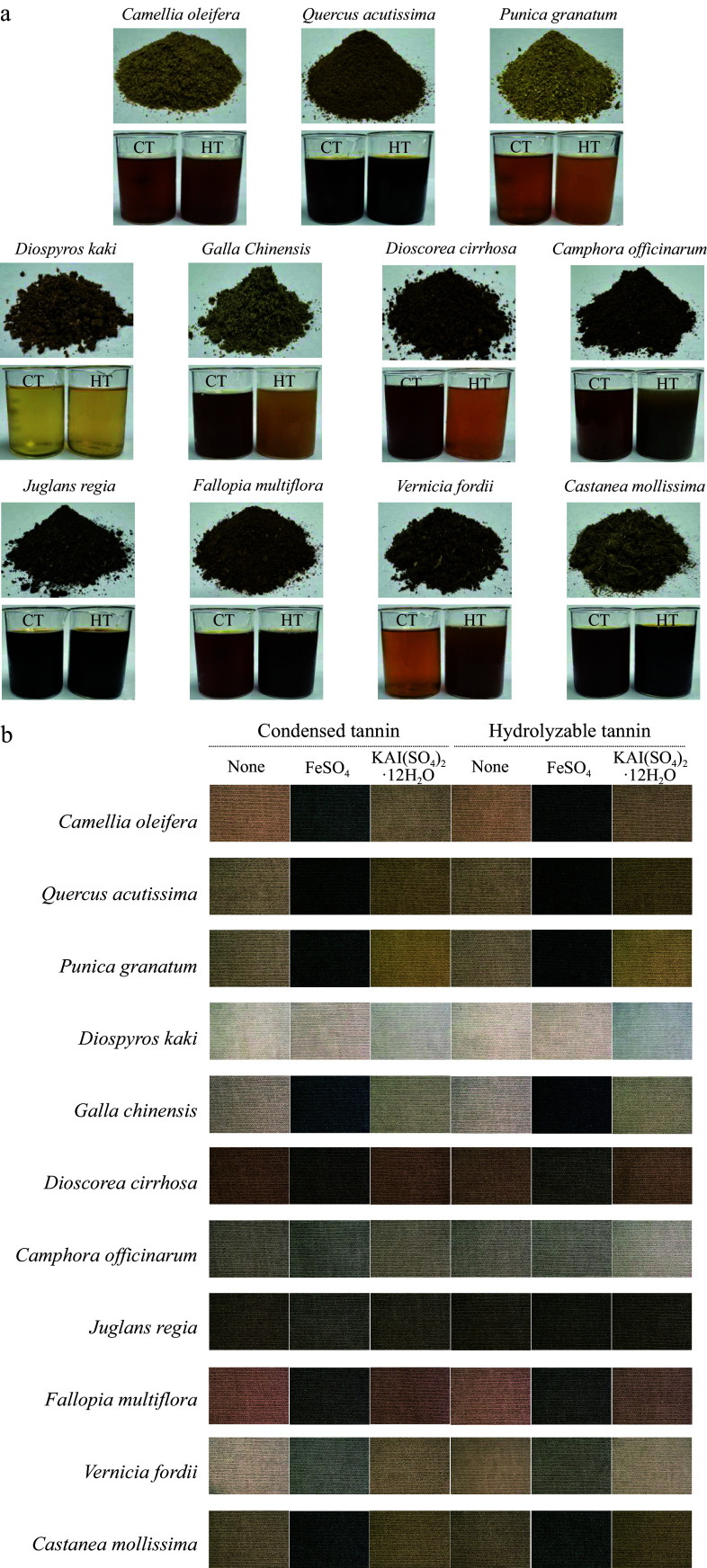
Plant dyes and dyeing results. (a) Photos of the powder and extracted dye solutions from the 11 plant dyes. CT refers to the dye solution extracted using 60% ethanol as the solvent, with condensed tannins as the main component; while HT refers to the dye solution extracted using water as the solvent, with hydrolysable tannins as the main component. (b) Dyeing results of cotton fabrics with different plant dyes and mordants.

Polyphenols, as natural dyes, are widely used because they can impart a variety of colors to textiles^[44]^. Tea leaves are rich in tea polyphenols that can provide fabrics with brown or black tones^[45]^; madder contains anthraquinone polyphenols, such as alizarin, primarily used in red dyes^[46]^; onion skins are rich in polyphenols such as quercetin, are used for yellow dyes^[47]^. The specific impact of key components on dyeing performance has not yet been fully evaluated. The type and concentration of polyphenols vary across different plants, and these polyphenol compounds may exhibit different dyeing effects during dyeing processes. Here, dye solutions from 11 polyphenol-rich plants were prepared using MAE with water and 60% anhydrous ethanol as solvents (Fig. 3a). Different solvents can result in the extraction of different primary components from plants. For example, when using water as the solvent, mostly hydrolyzable tannins (HTs) are extracted whereas 60% anhydrous ethanol extracts mainly condensed tannins (CTs). In addition, cotton fabrics were dyed with various mordants. The dyeing results, color yield (K/S), and CIE L*a*b* values are shown in Supplementary Table S1. First, the impacts of different components on the results of direct dyeing were examined. HTs and CTs exhibit minimal differences in the direct dyeing cotton fabrics, generally resulting in similar shades with only slight variations (Fig. 3b); however, the dyeing results for *V. fordii* CTs and HTs differ considerably. In addition to the obvious color differences, the CIEL*a*b* values also reveal notable discrepancies, particularly in the L* value (Supplementary Table S1). The L* value for CT-dyed fabrics is 31.28, while for HT-dyed fabrics, the L* value is 54.02, with a difference of 20 between them. Thus, the difference in lightness is the primary reason for the color variations observed in cotton fabrics dyed with different *V. fordii* dye solutions.

Different mordants had a crucial impact on the dyeing results (Fig. 3b and Supplementary Table S1). When ferrous sulfate was employed as a mordant, the K/S values of the cotton fabrics increased, significantly enhancing the dyeing performance of these dye solutions. The HT dyeing solution from *P. granatum* showed the greatest increase in the K/S value, from 13.66 to 27.34. A higher K/S value indicates a deeper color on the cotton fabric; therefore, after mordanting with ferrous sulfate, the L*, a*, and b* values decreased. In contrast, when alum was used as the mordant, the K/S values exhibited variability, with some increasing and others decreasing, while the L*, a*, and b* values showed no significant changes. Among the 22 dye solutions, the K/S values for cotton fabrics decreased after dyeing in eight of them, with the most significant decrease observed in the HT dye solution from *C. officinarum*, which were reduced by 2.12. On the other hand, the greatest increase in K/S value was seen in the HT dye solution of *G. chinensis*, which increased by 12.27; however, this increase was still less than that achieved when using ferrous sulfate as the mordant (13.68). The results indicate that iron ions when used as a mordant, better coordinate with the natural dye and cotton fabric, effectively blocking water-soluble groups on the dye and enhancing its stability, thus increasing the K/S values of the fabric^[22,48]^. Consequently, ferrous sulfate is identified as an excellent mordant that plays a crucial role in the dyeing process using natural dyes.

By comparing the dyeing results of 11 polyphenol-rich plants, the dyeing properties of polyphenolic plant dyes were investigated. These dyes typically impart black and brown tones to cotton fabrics, while *D. cirrhosa* and *F. multiflora* provide subtle red variations (Fig. 3b). The addition of mordants enhances the range of shades produced by natural dyes, expanding the spectrum to include yellow and orange, thus offering a wider array of color options.

### Mordant selection has a considerable effect on the color fastness of textiles with natural dyes

Color fastness is an important indicator in dyeing evaluation, as it directly affects the durability, stability, and practicality of dyes on textiles. The low color fastness of textiles dyed with natural dyes presents a significant challenge in practically applying these dyes on cotton fabrics. The primary focus of color fastness in relation to natural dyes is on washing fastness and light fastness. The color fastness of cotton fabric samples dyed with different main components from 11 polyphenol-rich plants under the influence of various mordants is shown in Table 1. When ferrous sulfate is used as a mordant, the washing fastness of cotton fabrics dyed with almost all plant dyes improves. In particular, the dyeing effect on *P. granatum* peels and *J. regia* shells was improved significantly, and the washing fastness could be increased from Grade 3 to Grade 4 or 5 after adding ferrous sulfate as a mordant. In contrast, alum displayed a less significant impact on the washing fastness of plant-dyed cotton fabrics. Meanwhile, the light fastness of most plant dyes to cotton fabrics dyed with different mordants was stable and remained in the range of Grade 4 to 5, indicating that the light fastness was generally good and using ferrous sulfate or alum as mordants had less effect on it. For dyes such as *D. kaki* and *V. fordii* shells, washing fastness and light fastness on cotton fabrics exhibit high levels, independent of the mordants added. Overall, the choice of mordant affects the dyeing performance of different plant dyes significantly, with ferrous sulfate generally providing notable improvements in washing fastness.

**Table 1:** Fastness properties of dyed samples.

Plant	Main ingredients	Mordant	Washing fastness	Light fastness
*Camellia oleifera*	Condensed tannin	None	4	4/5
		FeSO4	4/5	4/5
		KAl(SO_4_)_2_·12H_2_O	4/5	4/5
	Hydrolysable tannin	None	4	5
		FeSO4	4	5
		KAl(SO_4_)_2_·12H_2_O	5	5
*Quercus acutissima*	Condensed tannin	None	3	5
		FeSO4	3	5
		KAl(SO_4_)_2_·12H_2_O	4	5
	Hydrolysable tannin	None	3/4	5
		FeSO4	3	5
		KAl(SO_4_)_2_·12H_2_O	5	5
*Punica granatum*	Condensed tannin	None	4/5	5
		FeSO4	5	5
		KAl(SO_4_)_2_·12H_2_O	4	5
	Hydrolysable tannin	None	4	5
		FeSO4	4	5
		KAl(SO_4_)_2_·12H_2_O	4/5	5
*Diospyros kaki*	Condensed tannin	None	4/5	4
		FeSO4	5	4
		KAl(SO_4_)_2_·12H_2_O	5	4
	Hydrolysable tannin	None	5	4
		FeSO4	5	4
		KAl(SO_4_)_2_·12H_2_O	5	4
*Galla Chinensis*	Condensed tannin	None	4	4/5
		FeSO4	5	4/5
		KAl(SO_4_)_2_·12H_2_O	4/5	5
	Hydrolysable tannin	None	4	4/5
		FeSO4	4	5
		KAl(SO_4_)_2_·12H_2_O	4	4-5
*Dioscorea cirrhosa*	Condensed tannin	None	4	3
		FeSO4	5	5
		KAl(SO_4_)_2_·12H_2_O	4-5	4
	Hydrolysable tannin	None	4-5	3
		FeSO4	4	4
		KAl(SO_4_)_2_·12H_2_O	5	3
*Camphora officinarum*	Condensed tannin	None	4/5	4
		FeSO4	4/5	4
		KAl(SO_4_)_2_·12H_2_O	5	4
	Hydrolysable tannin	None	5	4
		FeSO4	5	4
		KAl(SO_4_)_2_·12H_2_O	5	4/5
*Juglans regia*	Condensed tannin	None	4/5	4/5
		FeSO4	5	4
		KAl(SO_4_)_2_·12H_2_O	4/5	4
	Hydrolysable tannin	None	4	5
		FeSO4	5	4/5
		KAl(SO_4_)_2_·12H_2_O	4/5	5
*Fallopia multiflora*	Condensed tannin	None	5	4
		FeSO4	5	4
		KAl(SO_4_)_2_·12H_2_O	4	4
	Hydrolysable tannin	None	5	3
		FeSO4	4/5	4/5
		KAl(SO_4_)_2_·12H_2_O	5	4
*Vernicia fordii*	Condensed tannin	None	5	4/5
		FeSO4	4/5	4
		KAl(SO_4_)_2_·12H_2_O	5	4
	Hydrolysable tannin	None	4	4
		FeSO4	4/5	4
		KAl(SO_4_)_2_·12H_2_O	4/5	4
*Castanea mollissima*	Condensed tannin	None	4	4/5
		FeSO4	5	4/5
		KAl(SO_4_)_2_·12H_2_O	4	5
	Hydrolysable tannin	None	4	4
		FeSO4	4	4/5
		KAl(SO_4_)_2_·12H_2_O	5	4/5

### Plant dyes are gentle and do not affect the surface structure of cotton fabrics

**Figure 4:**
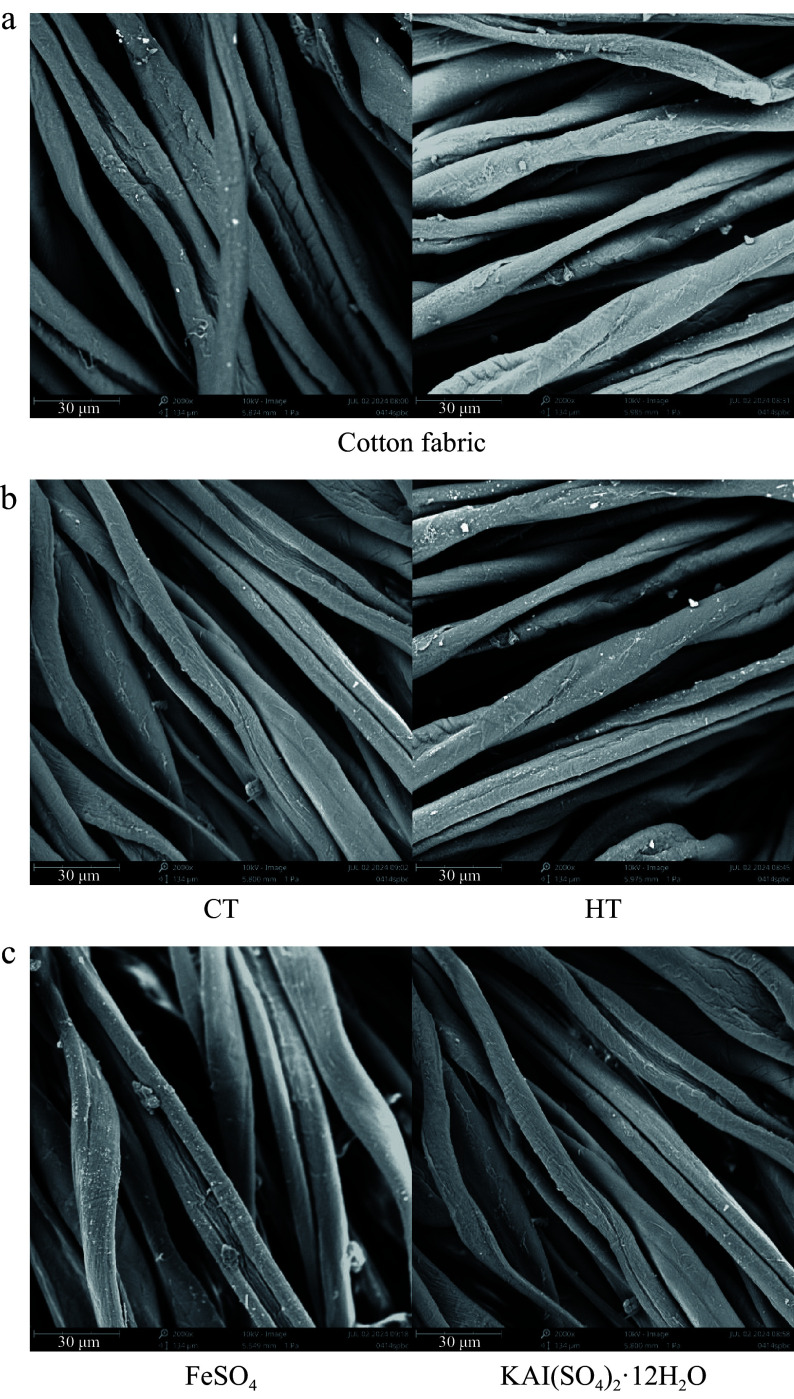
Surface morphology of cotton fabrics. (a) Surface morphology of untreated cotton fabric. (b) Surface morphology of cotton fabric directly dyed with different plant dye components from *Camphora officinarum* seeds. CT refers to the dye solution extracted using 60% ethanol as the solvent, with condensed tannins as the main component, while HT refers to the dye solution extracted using water as the solvent, with hydrolysable tannins as the main component. (c) Surface morphology of cotton fabric after post-mordanting with different mordants using plant dyes from *C. officinarum* seeds.

To visually assess the impact of plant dyes on cotton fabrics, SEM examinations were performed on dyed and undyed cotton fabric surfaces. Due to the large sample size, only a selection of the results are presented (Fig. 4). The undyed cotton fabrics displayed twisted fibril impressions along the fiber axis, characterized by relatively smooth surfaces. A close inspection of the images reveals no significant differences between dyed and undyed cotton fibers, whether with or without mordants. This observation underscores the gentle nature of plant-based natural dyes that, do not pose harm to the human body.

### Differences in the contents of specific compounds affect the dyeing performance of plant dyes on cotton fabrics significantly

**Figure 5:**
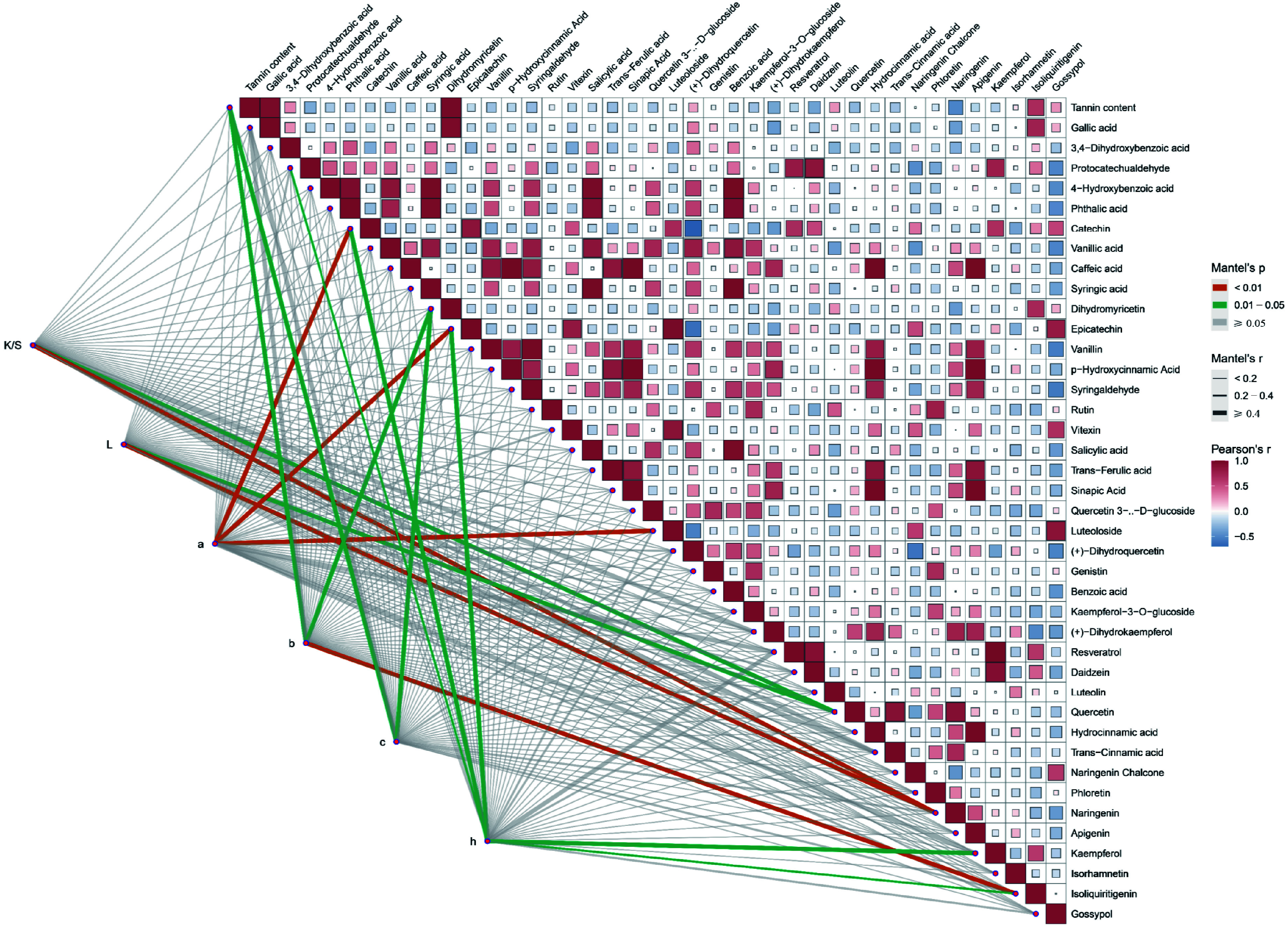
The content of polyphenols is considerably related to the dyeing performance of plant dyes on cotton fabrics. The Mantel test analysis was used to examine the correlation between CIE L*a*b* values and each of the 40 polyphenol metabolites. K/S represents the ratio of the dye absorption coefficient (k) to the dye scattering coefficient (s), L represents lightness, a represents the red-green coordinate, b represents the yellow-blue coordinate, c represents color saturation, and h represents hue. Orange lines indicate highly significant relationships (Mantel's *p* < 0.01), and green lines indicate significant relationships (0.01 ≤ Mantel's *p* < 0.05). The width of the lines corresponds to Mantel's r statistic, reflecting the respective distance correlations. Pairwise comparisons of polyphenol metabolites are shown, with a color gradient denoting Pearson's correlation coefficient.

The differences in compound content altered the composition of plant dyes, thus changing their dyeing effects on cotton fabrics. Variations in the amounts of specific compounds modified the composition of plant polyphenols, in turn affecting the dyeing process. To elucidate the roles of different compounds in dyeing, Pearson correlation analyses and Mantel test analyses were conducted based on targeted polyphenol metabolite detection data and dyeing performance results. The results indicated a correlation between the CIE L*a*b* values of dyed cotton fabrics and various polyphenolic compounds (Fig. 5). Specifically, the K/S and L* values were highly significantly correlated with naringenin (*p* < 0.01), and significantly correlated with quercetin (0.01 < *p* < 0.05). The a* value exhibited a highly significant correlation with catechin, epicatechin, and luteoloside (*p* < 0.01). Similarly, the b* value, similar to the c* value, was significantly correlated with tannin and dihydromyricetin (0.01 < *p* < 0.05), and also showed a highly significant correlation with isoliquiritigenin (*p* < 0.01). The h* value demonstrated significant correlations with five compounds protocatechualdehyde, catechin, epicatechin, kaempferol, and isoliquiritigenin (0.01 < *p* < 0.05). In conclusion, naringenin, epicatechin, catechin, dihydromyricetin, and tannin content significantly influenced the dyeing performance of cotton fabrics dyed with these 11 plant dyes.

### Innovative natural choice for plant dyes: naringenin

**Figure 6:**
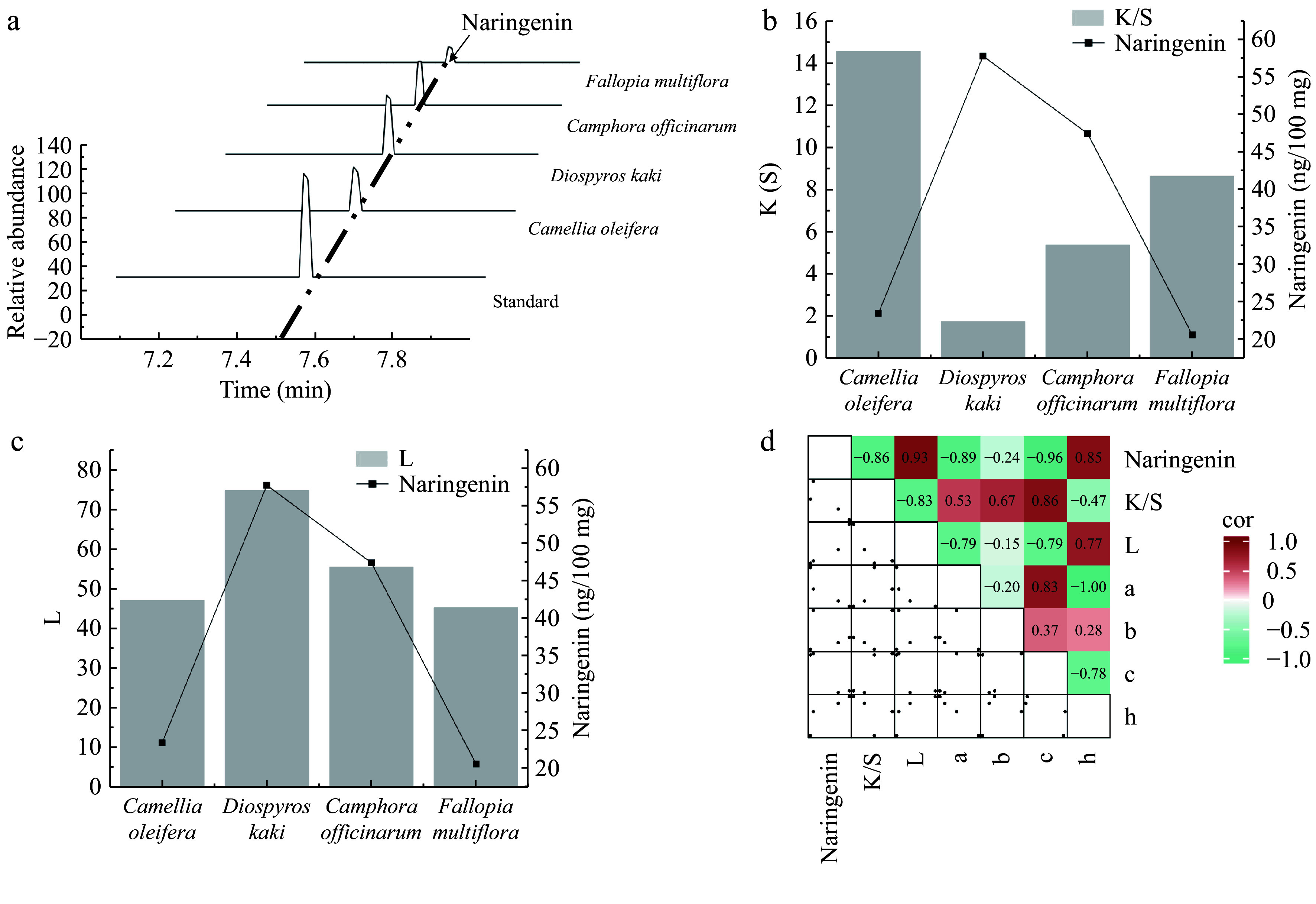
Analysis of the relationship between the naringenin content and dyeing performance. (a) Identification of naringenin in plants using high-performance liquid chromatography (HPLC). (b) Bar graph showing the correlation between the naringenin content and the ratio of the dye absorption coefficient (k) to the dye scattering coefficient (s) (K/S) value. (c) Bar graph showing the correlation between the naringenin content and lightness (L) value. (d) Heatmap illustrating the correlation between naringenin and dyeing performance. a represents the red-green coordinate, b represents the yellow-blue coordinate, c represents color saturation, and h represents hue.

Naringenin, the aglycone of naringin, is a natural polyphenolic compound known for its diverse biological activities, including anti-inflammatory, antioxidant, and antitumor properties. It is widely used in the pharmaceutical and food industries^[49]^. Through metabolomic analysis of 11 polyphenol-rich plants, naringenin was the only compound to show a highly significant correlation with the K/S and L values. Four plants with the highest naringenin content from these 11 plants, *D. kaki*, *C. officinarum* seeds, *C. oleifera* shells, and *F. multiflora*, were selected to investigate the specific effects of naringenin on cotton fabric dyeing further. Based on the peak graph, *D. kaki* contains the highest amount of naringenin, followed by *C. officinarum* seeds, then *C. oleifera* shells, with *F. multiflora* having the lowest content (Fig. 6a). The naringin content shows a significant negative correlation with the K/S value of dyed cotton fabric (R^2^ = −0.86) (Fig. 6b). In comparison, it has a significant positive correlation with the L* value of the dyed cotton fabric (R^2^ = 0.93) (Fig. 6c). In addition, naringenin showed a certain correlation with other dyeing performance values such as a, b, c, and h, further indicating its important role in the dyeing process (Fig. 6d). Thus, in future applications of plant dyes, the optimal amount of naringenin to balance the K/S and L* values for the best dyeing effect can be explored. The discovery of naringenin paves the way for improved plant dyes and an expansion in new fields for plant dyes.

### Sustainable innovation in plant dyes: the waste economy

**Figure 7:**
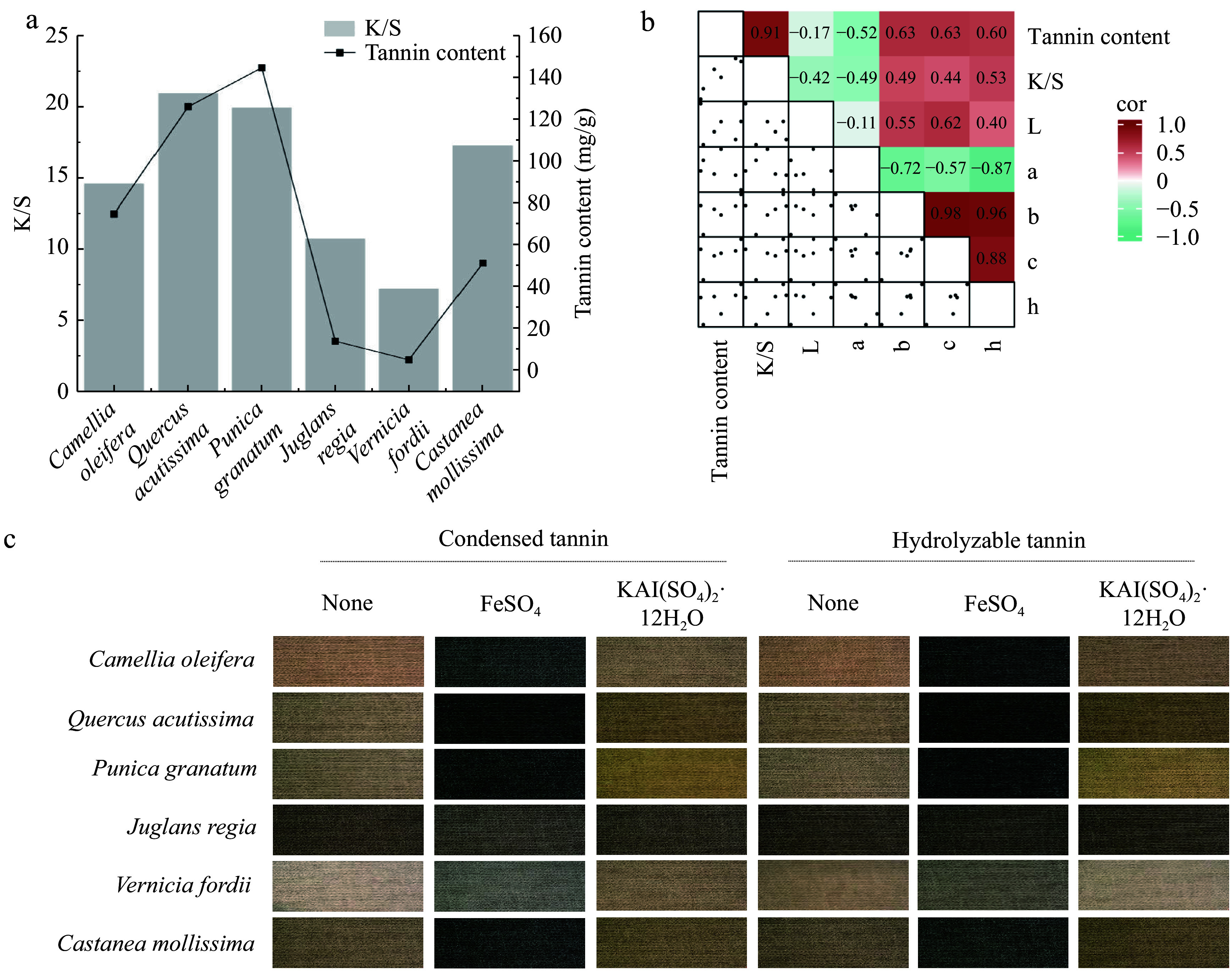
Analysis of the relationship between tannin content and dyeing performance. (a) Bar graph showing the correlation between the tannin content in plant waste and the ratio of the dye absorption coefficient (k) to the dye scattering coefficient (s) (K/S) value. (b) Heatmap illustrating the correlation between the tannin content and dyeing performance. L represents lightness, a represents the red-green coordinate, b represents the yellow-blue coordinate, c represents color saturation, and h represents hue. (c) Tannin imparting diverse colors to cotton fabrics.

Despite the widespread attention plant-based dyes have garnered for their environmental and health benefits, their high cost remains a significant barrier to large-scale adoption; therefore, the utilization of plant waste, allows for the diversified use of waste materials and mitigates the drawback of high costs associated with plant-based dyes^[30]^. The application of six types of plant waste from *C. oleifera* shell, *Q. acutissima* shell, *P. granatum* peel, *J. regia* shell, *V. fordii* shell, and *C. mollissima* shell in cotton fabric dyeing has been researched. These six plant wastes are rich in tannins, which are among the most important types of plant dyes, predominantly producing brown and black colors. It was not difficult to find a highly positive correlation between tannin content and K/S values (R² = 0.91) alongside a significant effect on b, c, and h values (Fig. 7a & b). Notably, different tannin components and mordants can impart different colors to cotton fabrics (Fig. 7c). The cotton fabrics dyed with *J. regia* shells and *V. fordii* shells exhibited uniform color with minimal variation, staying within the brown and gray ranges, respectively. In contrast, the fabrics dyed with *P. granatum* peels showed the greatest color variation, ranging from yellow–brown to gray. Further exploration of tannin components and selecting different mordants can broaden the color options of tannin-based plant dyes, thus promoting their wider application.

## Discussion

This study systematically conducted a metabolomic analysis of 11 polyphenol-rich plants for the first time and explored the impact of their polyphenolic compounds on the dyeing performance of cotton fabrics. Although some research on polyphenolic natural dyes has been conducted, most studies focus on the overall dyeing effects of specific plants or dye categories, with a lack of in-depth analysis regarding the individual components. The results identified four polyphenolic compounds that play a key role in dyeing performance, with naringenin showing a significant impact on the K/S, and L* values. In addition, this study revealed the important role of mordants in the plant dyeing process and explored the impact of using different mordants on dyeing performance. Dissimilar to traditional dyeing studies, the dyeing effects were a point of focus in addition to conducting a detailed analysis of the factors influencing dyeing performance, proposing a strategy to expand the color spectrum and enhance dyeing quality through the rational selection of mordants. More importantly, plant dyes with high environmental potential were selected, and about half of the plants used for dye extraction were sourced from waste plant materials. This not only highlights the eco-friendliness of polyphenolic compounds as natural dyes but also offers new ideas for promoting the sustainable development of the plant dye industry.

Polyphenols represent a significant class of secondary metabolites produced during the secondary metabolism of plants, playing various critical roles in plants. They exhibit strong antioxidant, antibacterial, and antiviral biological activities while also regulating plant growth and development and helping plants cope with biotic and abiotic stresses^[2,3,6,44]^. In textile dyeing, polyphenols provide rich colors while also enhancing fabric functionality with antibacterial, UV protection, and antioxidant properties. Currently, research on natural dyes focuses largely on the overall dyeing performance of specific classes of compounds, with a lack of in-depth analysis of their components. This has led to an incomplete understanding of the mechanisms and potential benefits of natural dyes. Here, 11 polyphenol-rich plants were selected and targeted metabolite analysis of their polyphenols, including *C. oleifera* shell, *Q. acutissima* shell, *P. granatum* peel, *D. kak*, *G. Chinensis*, *D. cirrhosa*, *C. officinarum* seeds, *J. regia* shell, *F. multiflora*, *V. fordii* shell, and *C. mollissima* shell were conducted. After identifying the polyphenol components, the plant extracts were applied to dye cotton fabrics, and the metabolite results were correlated with the dyeing outcomes to explore the impact of polyphenolic compounds on the dyeing performance of cotton fabrics.

As living standards improve and environmental awareness increases, natural dyes are receiving heightened attention. Derived from natural sources, these dyes exhibit excellent biodegradability, rendering them environmentally friendly, and safe^[12,50,51]^. Research indicates that natural dyes not only produce unique and soft colors but also enhance the value of fabrics by imparting distinctive fragrances and improving properties such as mosquito repellency, antibacterial effects, and UV protection^[52,53]^. The application of natural dyes on cotton fabrics underscores their eco-friendly attributes and facilitates the complete naturalization of cotton textiles, aligning with contemporary pursuits of green and ecological living. Nevertheless, natural dyes present certain limitations, including poor reproducibility in dyeing, low extraction efficiency, insufficient color fastness, and limited color ranges. These challenges impede the industrial advancement of natural dyes. Consequently, comprehensive research and development of natural dyes and their application in dyeing cotton textiles are of paramount importance.

Naringenin, epicatechin, catechin, dihydromyricetin, and tannin considerably influence the dyeing performance of cotton fabrics dyed with these 11 plant dyes. Interestingly, naringenin was the only compound that showed a highly significant correlation with two staining parameters (K/S value and L* value). Naringenin is a flavonoid compound mainly found in citrus fruits with various functions such as antioxidant, anti-inflammatory, antibacterial and antiviral. As an important class of polyphenolic plant dyes, flavonoid plant dyes occupy an important position in textile dyeing because of their unique dyeing properties and potential health benefits. Flavonoid dyes predominantly exhibit yellow, orange, and brown colors; however, the use of different mordants can also produce green or red colors, greatly extending the dyeing range of plant dyes. In addition, many flavonoid compounds possess antimicrobial and UV-resistant properties, making fabrics dyed with flavonoid-based dyes highly suitable for outdoor clothing and medical textiles. Future research could further explore the components of natural dyes by thoroughly analyzing their chemical compositions and identifying new methods to enhance dyeing performance. Investigating the specific constituents of natural dyes could offer new avenues to address the challenges in developing natural dyes. This approach should aid in optimizing dyeing processes and may also reveal advantages unique to certain natural dye components in improving color fastness, color stability, and environmental sustainability^[54,55]^; therefore, focusing on the components of natural dyes is an approach to addressing the current limitations in the field, while also functioning as a crucial step forward in advancing the natural dye industry.

In this study, the two most common dyeing processes for plant dyes, direct dyeing, and post-mordanting, were selected. For the post-mordanting process, the most common metal mordants, FeSO_4_ and KAl(SO_4_)_2_·12H_2_O were used. The use of mordants plays a crucial role in plant dyeing; metal mordants can form complexes with a dye and cotton fabric, enhancing the dyeing performance of plant-based dyes^[51,56,57]^. Consequently, future research on plant dyeing should place greater emphasis on mordants. This approach has the potential to improve the dyeing performance of plant-based dyes and expand the range of color tones achievable with these dyes^[51,58−60]^. While metal mordants are cost-effective and can shorten the dyeing process, they pose potential environmental threats. If dyeing wastewater is improperly treated, it can lead to environmental pollution, contradicting the eco-friendly principles underlying the use of plant dyes. In contrast, biological mordants are emerging as a viable alternative and are gradually gaining attention^[42,57,61]^. Erdem İşmal et al. investigated the effects of dyeing wool fabrics using almond shells and mordanted the dyed fabrics with both natural mordants (rosemary, cypress, and acorn) and metal mordants (FeSO_4_, KAl(SO_4_)_2_·12H_2_O, CuSO_4,_ and K_2_Cr_2_O_7_). The results indicated that all mordanted samples exhibited good color fastness^[62]^. Shahmoradi Ghaheh and colleagues conducted natural dyeing of cotton fabrics using *Hibiscus sabdariffa*, focusing on comparing the effects of metal mordants (FeSO_4_, KAl(SO_4_)_2_·12H_2_O, and CuSO_4_) and natural mordants (tannic acid, pine cone, lemon peel, and sodium alginate) on dyeing performance. They found that biological mordants and metal mordants enhanced the dyeing performance of cotton fabrics equally^[63]^; therefore, future research on natural dyes should prioritize natural mordants to advance the natural dye industry toward more rapid green and sustainable development.

## Conclusions

In conclusion, the targeted metabolism of 11 polyphenol-rich plants was analyzed, and the dyeing effect of their extracts on cotton fabrics was evaluated. This study demonstrated that, compared with direct dyeing, incorporating mordants enhanced the dyeing performance of the plant dyes significantly and broadened the spectrum of color tones, thus achieving a wider array of color options. Additionally, it was identified that naringenin, epicatechin, catechin, dihydromyricetin, and tannin played pivotal roles in the dyeing performance of cotton fabrics treated with these plant-based dyes. Notably, naringenin was the only compound that exhibited a highly significant correlation with two critical dyeing indicators: the K/S value and the L* value. In addition, this study highlights the significant potential of plant waste as a dye. Of the 11 plant dyes selected, nearly half are derived from plant waste, demonstrating the eco-friendly properties of natural dyes and providing a solid theoretical foundation for the innovation of sustainable dyeing technologies.

## SUPPLEMENTARY DATA

Supplementary data to this article can be found online.

## Data Availability

All data generated or analyzed during this study are included in this published article and its supplementary information files.
