# Supplementary material for: Polyphenol metabolomics reveals the applications and prospects of polyphenol-rich plants in natural dyes
Source: For Res (Fayettev). 2024 Dec 19;4:e038. doi: 10.48130/forres-0024-0035 (PMC11727558; doi:10.48130/forres-0024-0035)
Supplement: Supplementary file 1 — Supplementary data to this article can be found online. [file forres-0024-0035-S1.pdf]

Supplemental Table S1. The color characteristic values of the dyed samples.

Plant	Main ingredients	Mordant	Color	L	a	b	c	h	K/S
Camellia oleifera	Condensed Tannin	none	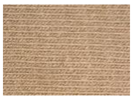	53.27	10.54	24.03	26.24	66.33	10.849
		FeSO4	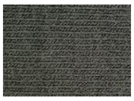	35.13	3.69	8.48	9.25	66.46	14.596
		$KAl(SO_4)_2 \cdot 12H_2O$	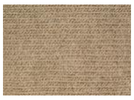	53.79	9.8	23.85	25.78	67.66	14.045
	Hydrolyzable Tannin	none	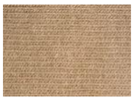	53.72	10.43	24.2	26.35	66.68	14.823
		FeSO4	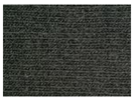	32.09	3.65	7.19	8.06	63.09	18.121
		$KAl(SO_4)_2 \cdot 12H_2O$	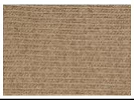	53.85	8.9	22.25	23.97	68.21	14.73
Quercus acutissima	Condensed Tannin	none	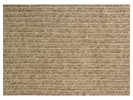	50.8	5.52	22.21	22.89	76.05	14.14
		FeSO4	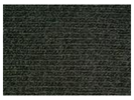	28.97	2.08	7.86	8.13	75.15	21.302
		$KAl(SO_4)_2 \cdot 12H_2O$	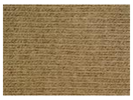	48.7	6.86	28.73	29.54	76.57	25.407
	Hydrolyzable Tannin	none	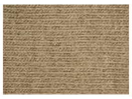	48.95	5.99	23.09	23.85	75.45	15.022
		FeSO4	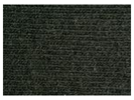	27.64	2.09	8.22	8.48	75.72	25.184
		$KAl(SO_4)_2 \cdot 12H_2O$	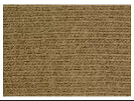	46.83	6.85	27.54	28.38	76.03	24.195
Punica granatum	Condensed Tannin	none	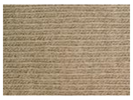	56.44	5.23	22.98	23.57	77.17	13.005
		FeSO4	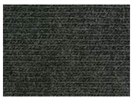	27.19	1.18	7.22	7.32	80.73	23.544
		$KAl(SO_4)_2 \cdot 12H_2O$	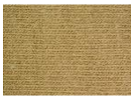	58.16	3.34	35.78	35.94	84.67	20.071
	Hydrolyzable Tannin	none	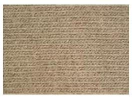	57.88	5.45	22.5	23.15	76.39	13.666

		FeSO4	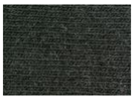	26.16	0.58	6.43	6.45	84.84	27.34
		$KAl(SO_4)_2 \cdot 12H_2O$	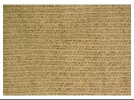	56.45	3.66	32.72	32.93	83.62	21.565
Diospyros kaki	Condensed Tannin	none	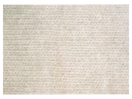	75	3.36	18.12	18.43	79.48	1.6738
		FeSO4	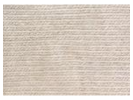	68.67	4.54	16.03	16.66	74.17	2.7305
		$KAl(SO_4)_2 \cdot 12H_2O$	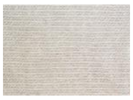	78.08	3.48	16	16.37	77.74	1.1172
	Hydrolyzable Tannin	none	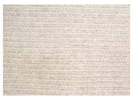	76.29	4.23	17.01	17.52	76.05	1.4186
		FeSO4	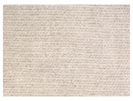	73.91	4.83	16.62	17.31	73.79	2.1806
		$KAl(SO_4)_2 \cdot 12H_2O$	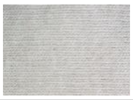	76.04	2.49	12.39	12.64	78.63	1.0874
Galla Chinensis	Condensed Tannin	none	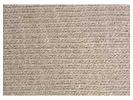	58.91	5.62	16.08	17.03	70.74	8.2839
		FeSO4	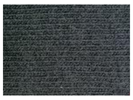	32.37	4.03	3.05	5.05	37.18	13.328
		$KAl(SO_4)_2 \cdot 12H_2O$	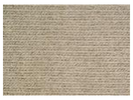	62.52	3.65	18	18.37	78.55	15.486
	Hydrolyzable Tannin	none	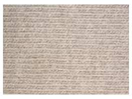	63.42	5.18	14.59	15.48	70.44	6.7983
		FeSO4	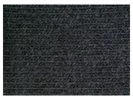	29.8	6.1	2.84	6.73	24.93	19.924
		$KAl(SO_4)_2 \cdot 12H_2O$	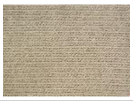	64.37	3.56	17.56	17.92	78.54	19.073
Dioscorea cirrhosa	Condensed Tannin	none	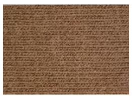	42.62	17.02	20.27	26.47	49.99	10.717
		FeSO4	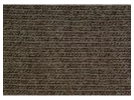	33.6	8.15	10.64	13.4	52.54	13.693
		$KAl(SO_4)_2 \cdot 12H_2O$	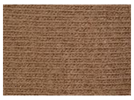	42.95	16.55	19.04	25.23	49.01	10.68

	Hydrolyzable Tannin	none	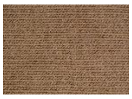	47.45	14.52	20.5	25.12	54.69	8.3984
		FeSO4	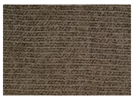	37.72	8.19	13.17	15.51	58.13	12.358
		$KAl(SO_4)_2\cdot 12H_2O$	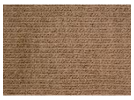	46.84	15.43	20.75	25.86	53.37	9.3221
Camphora officinarum	Condensed Tannin	none	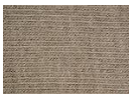	52.46	5.62	16.8	17.71	71.52	6.4603
		FeSO4	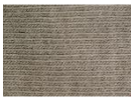	48.56	4.68	13.45	14.24	70.82	7.4734
		$KAl(SO_4)_2\cdot 12H_2O$	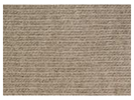	52.83	5.57	16.85	17.75	71.71	6.1538
	Hydrolyzable Tannin	none	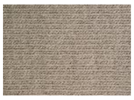	56.83	5.24	16.4	17.21	72.27	4.3837
		FeSO4	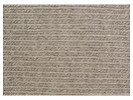	55.63	4.82	14.94	15.7	72.12	5.3382
		$KAl(SO_4)_2\cdot 12H_2O$	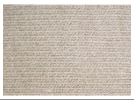	65.88	4.21	14.93	15.51	74.26	2.2581
Juglans regia	Condensed Tannin	none	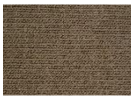	42.1	7.38	17.24	18.76	66.83	9.768
		FeSO4	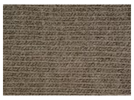	40.74	6.02	14.22	15.44	67.06	10.253
		$KAl(SO_4)_2\cdot 12H_2O$	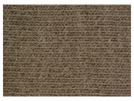	41.9	6.87	16	17.41	66.78	9.941
	Hydrolyzable Tannin	none	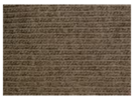	39.34	7.19	15.87	17.42	65.62	10.986
		FeSO4	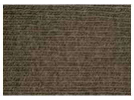	39.6	5.94	14.01	15.22	67.04	11.476
		$KAl(SO_4)_2\cdot 12H_2O$	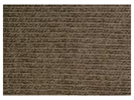	39.36	6.09	15.57	17.03	33.1	11.759
Fallopia multiflora	Condensed Tannin	none	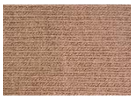	48.23	16.52	18.28	24.64	47.89	7.3458
		FeSO4	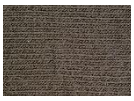	37.43	7.78	9.52	12.3	50.74	10.536

		$KAl(SO_4)_2\cdot 12H_2O$	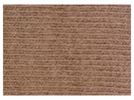	46.94	16.76	16.79	23.73	45.06	8.661
	Hydrolyzable Tannin	none	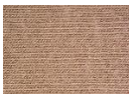	49.12	14.83	17.86	23.21	50.3	7.5218
		FeSO4	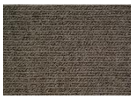	39.58	7.26	10.51	12.78	55.36	10.249
		$KAl(SO_4)_2\cdot 12H_2O$	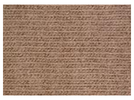	49.64	14.31	17.01	22.23	49.92	7.3151
Vernicia fordii	Condensed Tannin	none	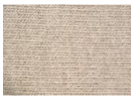	31.28	5.08	18.23	18.93	74.43	5.1417
		FeSO4	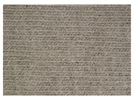	55.66	3.95	14.17	14.71	74.43	6.114
		$KAl(SO_4)_2\cdot 12H_2O$	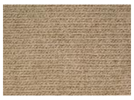	64.8	4.83	21.09	21.63	77.09	5.0237
	Hydrolyzable Tannin	none	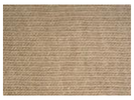	57.47	6.71	23.57	24.5	74.1	7.5573
		FeSO4	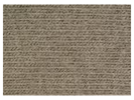	47.88	4.78	17.05	17.71	74.36	10.243
		$KAl(SO_4)_2\cdot 12H_2O$	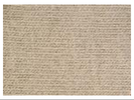	55.68	6.93	23.94	24.93	73.85	9.0091
Castanea mollissima	Condensed Tannin	none	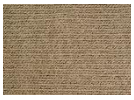	54.02	6.2	23.4	24.21	75.17	11.718
		FeSO4	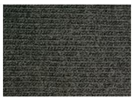	31.05	2.91	8.8	9.27	71.7	19.986
		$KAl(SO_4)_2\cdot 12H_2O$	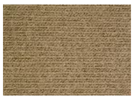	51.15	6.16	25.84	25.56	76.59	18.889
	Hydrolyzable Tannin	none	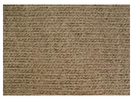	50.2	6.44	22.58	23.48	74.09	12.569
		FeSO4	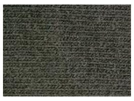	30.86	1.96	9.23	9.43	78.04	20.62
		$KAl(SO_4)_2\cdot 12H_2O$	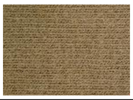	47.58	6.49	25.06	25.88	75.49	19.532
